# Structural basis for type VI secreted peptidoglycan dl-endopeptidase function, specificity and neutralization in *Serratia marcescens*

**DOI:** 10.1107/S0907444913022725

**Published:** 2013-11-19

**Authors:** Velupillai Srikannathasan, Grant English, Nhat Khai Bui, Katharina Trunk, Patrick E. F. O’Rourke, Vincenzo A. Rao, Waldemar Vollmer, Sarah J. Coulthurst, William N. Hunter

**Affiliations:** a Division of Biological Chemistry and Drug Discovery, College of Life Sciences, University of Dundee, Dundee DD1 5EH, Scotland; b Division of Molecular Microbiology, College of Life Sciences, University of Dundee, Dundee DD1 5EH, Scotland; c Centre for Bacterial Cell Biology, Institute for Cell and Molecular Biosciences, Newcastle University, Newcastle upon Tyne NE2 4HH, England

**Keywords:** amidases, cysteine proteases, disulfide linkage, effector, endopeptidases, Gram-negative, immunity protein, peptidoglycan, *Serratia marcescens*, type VI secretion system

## Abstract

Some Gram-negative bacteria target their competitors by exploiting the type VI secretion system to extrude toxic effector proteins. To prevent self-harm, these bacteria also produce highly specific immunity proteins that neutralize these antagonistic effectors. Here, the peptidoglycan endopeptidase specificity of two type VI secretion-system-associated effectors from *Serratia marcescens* is characterized. These small secreted proteins, Ssp1 and Ssp2, cleave between γ-d-glutamic acid and l-*meso*-diaminopimelic acid with different specificities. Ssp2 degrades the acceptor part of cross-linked tetratetrapeptides. Ssp1 displays greater promiscuity and cleaves monomeric tripeptides, tetrapeptides and pentapeptides and dimeric tetratetra and tetrapenta muropeptides on both the acceptor and donor strands. Functional assays confirm the identity of a catalytic cysteine in these endopeptidases and crystal structures provide information on the structure–activity relationships of Ssp1 and, by comparison, of related effectors. Functional assays also reveal that neutralization of these effectors by their cognate immunity proteins, which are called resistance-associated proteins (Raps), contributes an essential role to cell fitness. The structures of two immunity proteins, Rap1a and Rap2a, responsible for the neutralization of Ssp1 and Ssp2-like endopeptidases, respectively, revealed two distinct folds, with that of Rap1a not having previously been observed. The structure of the Ssp1–Rap1a complex revealed a tightly bound heteromeric assembly with two effector molecules flanking a Rap1a dimer. A highly effective steric block of the Ssp1 active site forms the basis of effector neutralization. Comparisons with Ssp2–Rap2a orthologues suggest that the specificity of these immunity proteins for neutralizing effectors is fold-dependent and that in cases where the fold is conserved sequence differences contribute to the specificity of effector–immunity protein interactions.

## Abstract 2

Crystal structures of type VI secretion system-associated immunity proteins, a peptidoglycan endopeptidase and a complex of the endopeptidase and its cognate immunity protein are reported together with assays of endopeptidase activity and functional assessment.

## 1. Introduction   

Specialized secretion systems are key to bacterial fitness, survival and pathogenesis. They perform a myriad of roles in the processes that influence growth, colonization, attack and defence as bacteria interact with each other and with eukary­otic organisms (Filloux, 2011 ▶; Gerlach & Hensel, 2007 ▶). The recently identified type VI secretion system (T6SS), which is present in about 25% of Gram-negative bacteria for which genome sequences are available (Boyer *et al.*, 2009 ▶), can be used to target bacterial and eukaryotic cells and is thus important for both inter-bacterial competition and pathogenesis (Burtnick *et al.*, 2011 ▶; de Pace *et al.*, 2010 ▶; Jani & Cotter, 2010 ▶). Antibacterial T6SSs mediate the efficient killing of competitors by direct injection of toxic antagonistic effector proteins into target cells (Murdoch *et al.*, 2011 ▶; Hood *et al.*, 2010 ▶; MacIntyre *et al.*, 2010 ▶; Schwarz *et al.*, 2010 ▶).

T6SS gene clusters encode the core components of a secretion machine capable of membrane perforation. This is a multi-protein needle-like assembly, resembling the contractile bacteriophage tail, that delivers effectors across three envelope layers in a single step (Bönemann *et al.*, 2010 ▶; Cascales & Cambillau, 2012 ▶; Silverman *et al.*, 2012 ▶). The clusters also encode accessory and post-translational regulatory components. Some T6SS-secreted effector proteins are also encoded within these large gene clusters. In the case of antibacterial T6SSs, effectors are always encoded adjacent to specific cognate immunity proteins. Immunity proteins bear appropriate signals to direct their localization to the compartment in which the toxic effectors act; for example, a Sec signal sequence guides localization to the periplasm. The provision of a cognate immunity protein provides protection against attack from sister cells (Coulthurst, 2013 ▶).

Different catalytic activities are associated with T6SS effectors. These include actin cross-linking and ADP-ribosyl­ation, which disrupt the cytoskeletons of mammalian and amoebal cells (Pukatzki *et al.*, 2007 ▶; Rosales-Reyes *et al.*, 2012 ▶), and phospholipases, which degrade phosphatidyl­ethanolamine, the major component of the bacterial membrane (Russell *et al.*, 2013 ▶). The best-characterized effectors are peptidoglycan hydrolases, which exhibit potent antibacterial activity (Russell *et al.*, 2011 ▶, 2012 ▶). These enzymes degrade peptidoglycan, the heteropolymer that occupies the periplasmic space, imparts mechanical strength to the cell wall and helps to maintain the shape of Gram-negative bacteria.

Peptidoglycan hydrolases constitute a large enzyme family which displays a rich diversity in terms of structure, mechanism and specificity (Vollmer *et al.*, 2008 ▶). There are enzymes specific for every glycosidic and amide bond in peptidoglycan. Such diversity is exploited to regulate bacterial cell growth, division and daughter-cell separation and, of particular interest here, to provide bactericidal properties that can be exploited in niche competition. Several classes of peptidoglycan hydrolases have been identified as T6SS effector proteins. These are termed Tse proteins. Tse3 from *Pseudomonas aeruginosa* is a muramidase, cleaving the glycan backbone (Russell *et al.*, 2011 ▶), and the C-terminal domain of *Vibrio cholerae* VgrG-3 has been suggested to have lysozyme-like muramidase activity (Brooks *et al.*, 2013 ▶). A diverse group of T6SS-secreted peptidoglycan amidases which cleave peptide cross-links has been described (Russell *et al.*, 2012 ▶). Within this superfamily, four distantly related families with distinct cleavage specificities were defined. Family 1 (Tae1), which includes Tse1 from *P. aeruginosa*, hydrolyses peptide cross-links at the γ-d-glutamyl-*meso*-diaminopimelate dl-bond, representatives of families 2 and 3 (Tae2 and Tae3) hydrolyse dd-cross-links between d-*m*A_2_pm (*meso*-diaminopimelate) and d-alanine, and a representative of family 4, Tae4 from *Salmonella enterica* serovar Typhimurium, also hydrolyses the γ-d-glutamyl-*m*A_2_pm dl-bond. Structures of Tse1 from *P. aeruginosa* and of Tae4 from *Salmonella* Typhimurium and *Enterobacter cloacae* (Benz *et al.*, 2012 ▶; Chou *et al.*, 2012 ▶; Ding *et al.*, 2012 ▶; Zhang *et al.*, 2013 ▶) place these T6-secreted effectors in the NlpC/P60 family of endopeptidases, amidases and acyltransferases (named after the new lipoprotein C from *Escherichia coli* and a 60 kDa extracellular protein from *Listeria monocytogenes*; Anantharaman & Aravind, 2003 ▶). The opportunistic pathogen *Serratia marcescens* has recently been shown to utilize T6-dependent secretion of two family 4 amidases, Ssp1 and Ssp2, to mediate antibacterial activity (English *et al.*, 2012 ▶).

Bacteria that secrete potent peptidoglycan hydrolase effectors using the T6SS to attack competitors could generate a deleterious effect on their own population. To cope with this potential for friendly-fire damage, such bacteria also possess cognate immunity or resistance proteins located in the periplasm. These immunity proteins bind their cognate effectors with low nanomolar affinity to neutralize them in a highly specific manner (English *et al.*, 2012 ▶). Four distinct families of putative immunity proteins are associated with the four Tae amidase families (Tai1–Tai4; Russell *et al.*, 2012 ▶). In *S. marcescens*, the resistance-associated proteins Rap1a and Rap2a neutralize Ssp1 and Ssp2, respectively (English *et al.*, 2012 ▶). Additionally, two other Rap proteins, Rap1b and Rap2b, are encoded together with Ssp1 and Ssp2 in the same locus within the T6SS gene cluster. The structures of Rap1b and Rap2b revealed a novel α-helix fold and a dimeric assembly (English *et al.*, 2012 ▶), which was later observed in the Tai4 proteins from *E. cloacae* (*Ec*Tai4) and *Salmonella* Typhimurium (*S*TTai4; Zhang *et al.*, 2013 ▶). This fold is a template for some T6SS immunity proteins, called Tsi proteins, but not all. For example, analysis of the Tse1/Tsi1 effector/immunity protein combination found in *P. aeruginosa* revealed Tsi1 to be an all-β protein (Benz *et al.*, 2012 ▶; Ding *et al.*, 2012 ▶; Shang *et al.*, 2012 ▶), whereas Tsi2, the immunity protein associated with the cytoplasmic effector Tse2, in the same organism has a helical fold that is distinct again (Li *et al.*, 2012 ▶). That effector immunity defence systems based on distinct folds have evolved is perhaps to be expected, given the strong evolutionary pressure applied by multifarious secreted effectors. Indeed, such pressure may even have contributed to the acquisition of effector–immunity pairs encoded outside T6SS operons (for example, all three Tse/Tsi pairs in *P. aeruginosa*).

Here, we assess the peptidoglycan hydrolase specificity of Ssp1 and Ssp2 and the protective role of cognate immunity proteins. We define the specificity of the enzymes and confirm using site-directed mutagenesis that an *in vivo* cell-killing mechanism is directly attributable to their catalytic activity. We report crystallographic analyses of *S. marcescens* Ssp1, a disabled mutant (Ssp1-C50A), Rap1a, Rap2a and the heterotetrameric Ssp1–Rap1a complex. Our data provide information on the enzyme mechanism, aspects of substrate specificity, the structural classification of Ssp1 and Rap proteins, including the identification of a novel immunity protein fold, and the molecular details of how an effector is neutralized by its cognate immunity protein, and suggest generic features related to function that allow the classification of these proteins into distinct groups. Finally, we consider diversity within the Tae4 family of effectors and their immunity proteins and how this may explain the presence of multiple homologues within the same organism.

## 2. Materials and methods   

### 2.1. Recombinant protein production and effector–immunity protein complex formation   

Recombinant Ssp1 and Ssp2 were expressed in *E. coli* BL21 (DE3), and Rap1a and Rap2a, minus their N-terminal periplasmic targeting sequences, were expressed in *E. coli* Rosetta-gami (DE3) and purified in high yield using established protocols (English *et al.*, 2012 ▶). The predicted catalytic cysteine of Ssp1 and Ssp2 (Cys50) was mutated to an alanine using the QuikChange II Site-Directed Mutagenesis Kit (Stratagene) and the altered proteins were purified as for the wild-type samples. For co-expression of the Ssp1–Rap1a and Ssp2–Rap2a complexes, full-length mature Rap1a (residues 25–127) and Rap2a (residues 25–131), without signal peptides, were cloned using *Nde*I and *Xho*I restriction sites at the second multiple-cloning site of the co-expression vector pACYCDuet-1 (Novagen), and full-length Ssp1 (residues 1–163) and Ssp2 (residues 1–158) were subsequently cloned using *Nco*I and *Bam*HI restriction sites at the first multiple-cloning site. This introduces an N-terminal His_6_ tag into the expressed product. Both the Ssp1–Rap1a and Ssp2–Rap2a complexes were produced in *E. coli* Rosetta-gami (DE3) and were purified by immobilized metal-ion affinity chromatography (English *et al.*, 2012 ▶) and size-exclusion gel-filtration chromatography with a Superdex 75 10/300 GL column (GE Healthcare). The samples were concentrated to 13.5 mg ml^−1^ by centrifugation (10 000 molecular-weight cutoff, Amicon) and dialyzed into 10 m*M* sodium phosphate pH 6.4. A high level of purity of greater than 95% was confirmed by SDS–PAGE. Size-exclusion chromatography was also used to investigate the association of cognate Ssp-C50A mutant–Rap combinations, with the proteins being mixed in equimolar amounts prior to separation, as described by English *et al.* (2012 ▶).

### 2.2. Peptidoglycan-cleavage assay   

Purified peptidoglycan sacculi (300 µg) from *E. coli* D456, consisting mainly of tetrapeptides with lower fractions of tripeptides and pentapeptides (Chou *et al.*, 2012 ▶), were incubated with either Ssp1 or Ssp2 (100 µg ml^−1^) or, as a control, no enzyme in 300 µl 20 m*M* sodium phosphate pH 4.8 for 4 h at 310 K. The samples were incubated with 40 µg ml^−1^ of the muramidase Cellosyl (kindly provided by Höchst AG, Frankfurt, Germany) for 16 h at 310 K to convert the residual peptidoglycan and solubilized fragments into muropeptides. The sample was boiled for 10 min and insoluble material was removed by centrifugation. The muropeptides were reduced with sodium borohydride and analyzed by high-pressure liquid chromatography using established methods (Glauner, 1988 ▶; Chou *et al.*, 2012 ▶; Russell *et al.*, 2012 ▶). The profile/retention times were compared with previous samples for which mass-spectrometric analyses with fragmentation had been carried out and which had identified that the linear disaccharide hexapeptide elutes before the disaccharide dipeptide, whereas the branched disaccharide hexapeptide elutes after the disaccharide dipeptide.

### 2.3. Phenotypic assays to characterize the C50A mutants of Ssp1 and Ssp2   

These assays were performed using the bacterial strains, plasmids and protocols described previously (English *et al.*, 2012 ▶). The QuikChange II Site-Directed Mutagenesis Kit (Stratagene) was used to generate C50A (Cys50 to Ala) mutants of Ssp1 and Ssp2 in the existing plasmids pSC152 and pSC138 (for periplasmic expression in *E. coli*) and pSC539 and pSC541 (for complementation of the cognate mutation in *S. marcescens*). Microscopic analysis, antibacterial co-culture (competition) assays and immunodetection of secreted Ssp2 were performed as described in the previous study. In brief, the format of the antibacterial co-culture assays was that the attacker and target strains were mixed in a 1:1 ratio, co-cultured on solid Luria–Bertani (LB) media for 7.5 h at 303 K and the surviving target cells (in this case a streptomycin-resistant version of the Δ*rap2a*, Δ*clpV* mutant) were enumerated by serial dilution and viable counts on streptomycin-containing media. ClpV is an ATPase that is essential for the type VI secretion system to function and so deletion provides an appropriate control. Statistical significance testing was performed using ANOVA followed by Dunnett’s post-test (GraphPad Prism software). For the detection of Ssp1 and Ssp2 levels in solid-grown *E. coli* or *S. marcescens*, cells were removed from the surface of agar plates, resuspended in liquid media, boiled and the total cell extract was separated by SDS–PAGE followed by anti-Ssp1 or anti-Ssp2 immunoblotting as described previously (English *et al.*, 2012 ▶).

### 2.4. Bioinformatic analyses   

Multiple Ssp/Tae4 homologues were identified from public databases as part of previous studies (English *et al.*, 2012 ▶; Russell *et al.*, 2012 ▶). Multiple sequence alignments were generated using *MUSCLE* (Edgar, 2004 ▶), and *Jalview* (Waterhouse *et al.*, 2009 ▶) was used to visualize the alignment and to calculate the resulting tree (using neighbour-joining construction and the BLOSUM62 distance matrix). Genomic analyses using publicly available databases (http://www.ncbi.nlm.nih.gov/ and http://www.sanger.ac.uk/resources) allowed the identification of adjacently encoded candidate immunity proteins, which were then used as bait to interrogate the *S. marcescens* Db11 genome and determine the Rap protein to which each was most closely related.

### 2.5. Crystallographic analyses   

#### 2.5.1. Crystal growth and data collection   

For crystallization trials, Rap1a was dialyzed against 25 m*M* Tris–HCl, 150 m*M* sodium chloride pH 7.5 and all other samples were in 100 m*M* sodium phosphate pH 6.4. The sitting-drop vapour-diffusion method was used with 0.2 µl drops with a 1:1 ratio of protein solution to reservoir solution at 293 K. Several commercially available screens were used in 96-well plates with a Phoenix Liquid Handling System (Rigaku, Art Robbins Instruments) to scout out initial conditions, which were then optimized.

Crystals of Ssp1 were obtained by combining protein solution at a concentration of 10 mg ml^−1^ with reservoir solution consisting of 0.2 *M* potassium sulfate, 20% PEG 3350. Orthorhombic block crystals grew to a maximum dimension of approximately 350 µm over 5 d. The Ssp1-C50A mutant (10 mg ml^−1^) gave isomorphous crystals (maximum dimension of 250 mm) in 2 d using reservoir solution consisting of 0.1 *M* sodium citrate pH 5.5, 20% PEG 3000. The Ssp1–Rap1a complex at 13.5 mg ml^−1^ formed clusters of plate-like crystals using a reservoir solution consisting of 12.5% PEG 1000, 12.5% PEG 3350, 12.5% MPD. These crystals attained a maximum size of 200 µm within 3 d. A single-crystal fragment was removed from the cluster for diffraction measurements. Monoclinic blocks of Rap2a were grown by combining a protein concentration of 13.5 mg ml^−1^ with a reservoir solution consisting of 25% PEG 1000, 0.1 *M* MES pH 6.5. These crystals attained a maximum dimension of 200 µm within 5 d. A slender ortho­rhombic crystal of Rap1a with approximate dimensions of 150 × 35 × 35 µm was observed after about one month using a reservoir solution consisting of 25% PEG 3350, 100 m*M* bis-tris pH 5.5. We were unable to obtain crystals of the Ssp2–Rap2a complex.

All crystals were soaked briefly in mother liquor adjusted to contain 20%(*v*/*v*) glycerol as a cryoprotectant prior to flash-cooling in liquid nitrogen and use in diffraction experiments. Data for Ssp1, the Ssp1-C50A mutant and the Ssp1–Rap1a complex were measured at 100 K using a Rigaku MicroMax-007 rotating-anode X-ray generator (Cu *K*α) coupled to a Saturn 944 CCD detector and were processed using *XDS* (Kabsch, 2010 ▶) or *HKL*-3000 (Minor *et al.*, 2006 ▶). The Rap1a and Rap2a data were collected on beamlines I04 and I03 of the Diamond Light Source, respectively, and were indexed and integrated in *iMosflm* (Battye *et al.*, 2011 ▶). Data sets were analyzed and scaled with *POINTLESS* and *SCALA* (Evans, 2006 ▶) from the *CCP*4 program suite (Winn *et al.*, 2011 ▶).

The isomorphous Ssp1 and Ssp1-C50A crystals contained two polypeptides in the asymmetric unit with an estimated solvent content of 50% and a *V*_M_ of 2.46 Å^3^ Da^−1^. The Ssp1–Rap1a complex crystal presented a heterodimer in the asymmetric unit with an estimated solvent content of 40% and a *V*_M_ of 2.06 Å^3^ Da^−1^. Rap2a crystallized with four molecules in the asymmetric unit, an estimated solvent content of 40% and a *V*_M_ of 2.06 Å^3^ Da^−1^, whilst Rap1a displayed two molecules in the asymmetric unit with an estimated solvent content of 45% and a *V*_M_ of 2.06 Å^3^ Da^−1^.

#### 2.5.2. Structure determination and refinement   

The Ssp1 structure was solved targeting the single-wavelength anomalous scattering properties of sulfur (Micossi *et al.*, 2002 ▶). *Auto-Rickshaw*, the EMBL Hamburg automated structure-determination platform (Panjikar *et al.*, 2005 ▶), was used. The heavy-atom structure-factor contributions were estimated in *SHELXC* (Sheldrick, 2010 ▶; Sheldrick *et al.*, 2001 ▶) and the maximum resolution for substructure determination and initial phase calculation was set to 2.30 Å. All 14 heavy atoms (sulfurs) were found using *SHELXD* (Schneider & Sheldrick, 2002 ▶). The correct hand for the substructure was determined using *ABS* (Hao, 2004 ▶) and *SHELXE* (Sheldrick, 2002 ▶) and the initial phases produced a CC (correlation coefficient) of 0.32. The initial phases were improved by density modification (Terwilliger, 2003 ▶) prior to phase extension to 1.85 Å resolution. The CC improved to 0.63, resulting in an electron-density map with excellent quality. Almost 80% of the model was constructed in *ARP*/*wARP* (Langer *et al.*, 2008 ▶). The initial model consisted of two polypeptides of 295 residues in total, with *R*_work_ and *R*_free_ values of 29.7 and 31.4%, respectively. Subsequent model building extended this to 326 residues, with the *R*_work_ and *R*_free_ values improving to 20.7 and 24.1%, respectively.

A molecule of Ssp1 was used to solve the structures of the Ssp1–Rap1a complex and the Ssp1-C50A mutant by molecular replacement (*autoMR*; Winn *et al.*, 2011 ▶). The structures of Rap1a and Rap2a were solved by molecular replacement (*Phaser*; McCoy *et al.*, 2007 ▶) using the structure of Rap1a from the Ssp1–Rap1a complex and Rap2b (PDB entry 4b6i; English *et al.*, 2012 ▶), respectively. In the latter case the sequence identity shared by the search model and the target structure is only about 20%.

All structures were refined in an iterative process combining *REFMAC*5 (Murshudov *et al.*, 2011 ▶) with electron-density and difference-density map inspections and model manipulations in *Coot* (Emsley *et al.*, 2010 ▶). For those structures with multiple copies in the asymmetric unit, tight NCS (noncrystallographic symmetry) restraints were imposed which were gradually released during refinement. Translation/libration/screw analysis (TLS) refinements were applied with the appropriate groups determined using the *TLSMD* server (Painter & Merritt, 2006 ▶). Water molecules, and in the case of the Ssp1 structure also potassium and sulfate ions, were added during the refinement process. Where appropriate, dual rotamer side-chain conformations were also included. Refinements were terminated when there were no significant changes in the *R*_work_ and *R*_free_ values and inspection of the difference density maps suggested that no further corrections or additions were justified.

*MolProbity* (Chen *et al.*, 2010 ▶) was used to investigate the model geometry. Secondary-structure and surface-interaction analyses were performed using *DSSP* (Kabsch & Sander, 1983 ▶) and *PISA* (Krissinel & Henrick, 2007 ▶), respectively. Figures were prepared using *ALINE* (Bond & Schüttelkopf, 2009 ▶) and *PyMOL* (Schrödinger). The *DALI* server was used to search the PDB for structural homologues, whilst superpositions were calculated using *DaliLite* (Holm & Park, 2000 ▶). Relevant crystallographic statistics and geometric details of the refined models were extracted from the programs used in the analyses and are reported in Table 1 ▶.

## 3. Results and discussion   

### 3.1. Activity of Ssp1 and Ssp2 and neutralization by cognate Rap partners   

**Figure 1:**
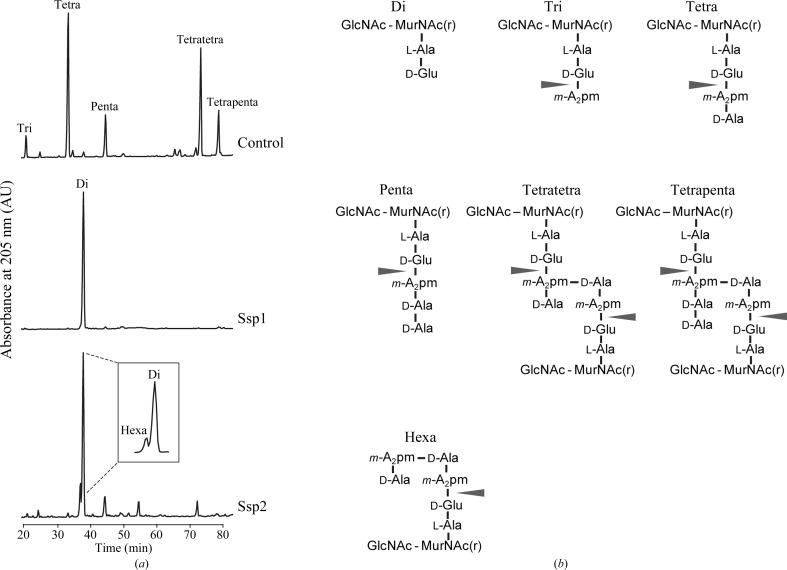
The specificity of Ssp1 and Ssp2. (*a*) Peptidoglycan from *E. coli* strain D456 was incubated with Ssp1, Ssp2 or no enzyme (control) followed by digestion with the muramidase Cellosyl and analysis of the resulting muropeptides by HPLC. Both Ssp1 and Ssp2 cleaved non-cross-linked (tri, tetra and penta) and cross-linked (tetratetra and tetrapenta) muropeptides between d-glutamate and *meso*-diaminopimelic acid (*m*-A_2_pm), resulting in the disaccharide dipeptide (di) product. Ssp2 also produced a small amount of the disaccharide hexapeptide (hexa) product. (*b*) Proposed structures of the muropeptides separated in (*a*). The arrows indicate the cleavage sites of d-*i*Glu-*m*-A_2_pm endopeptidases in peptidoglycan. GlcNAc, *N*-acetylglucosamine; MurNAc(r), *N*-acetylmuramitol; *m*-A_2_pm, *meso*-diaminopimelic acid.

We previously identified *S. marcescens* Ssp1 and Ssp2 as periplasmic acting antibacterial T6SS effectors (English *et al.*, 2012 ▶) and noted their membership, on the basis of sequence similarity, of the family 4 amidases (Tae4 proteins) proposed by Russell *et al.* (2012 ▶). We aimed to experimentally determine the enzymatic activity of each protein. The incubation of peptido­glycan sacculi with purified Ssp1 or Ssp2, followed by muramidase digestion and high-pressure liquid-chromatography analysis, revealed the disaccharide dipeptide as the single major product. This indicates that both enzymes cleave the amide bond in peptidoglycan between isoglutamic acid and *meso*-diaminopimelic acid. Ssp1 quantitatively hydrolyzed monomeric tripeptides, tetrapeptides and pentapeptides, as well as dimeric tetratetrapeptides and tetrapentapeptides, on both the acceptor and the donor side (Fig. 1 ▶). In the cross-linked peptides, the ∊-amino group of the *meso*-diamino­pimelic acid residue at position 3 on the acceptor side is connected by an amide bond to the α-carboxylic group of d-­alanine at position 4 on the donor side. Although Ssp2 was active against these muropeptides, the cleavage of monomeric pentapeptide and dimeric tetratetrapeptide was less efficient and the partially cleaved disaccharide hexapeptide product was detected (Fig. 1 ▶ *a*). This behaviour is similar to the family 4 amidase Tae4 from *Salmonella* Typhimurium (*S*TTae4, also known as Tae4^TM^; Russell *et al.*, 2012 ▶). Hence, Ssp2 and *S*TTae4 preferentially cleave tetrapeptides in the acceptor part of cross-linked peptides. This is distinct from *P. aeruginosa* Tse1, which preferentially cleaves pentapeptides in the donor part of cross-linked peptides (Chou *et al.*, 2012 ▶; Russell *et al.*, 2012 ▶). These data indicate that Ssp1 and Ssp2 are distinctive peptido­glycan dl-endopeptidases in terms of specificity, with the former being more promiscuous regarding the chemical structure that it is able to recognize and then cleave.

**Figure 2:**
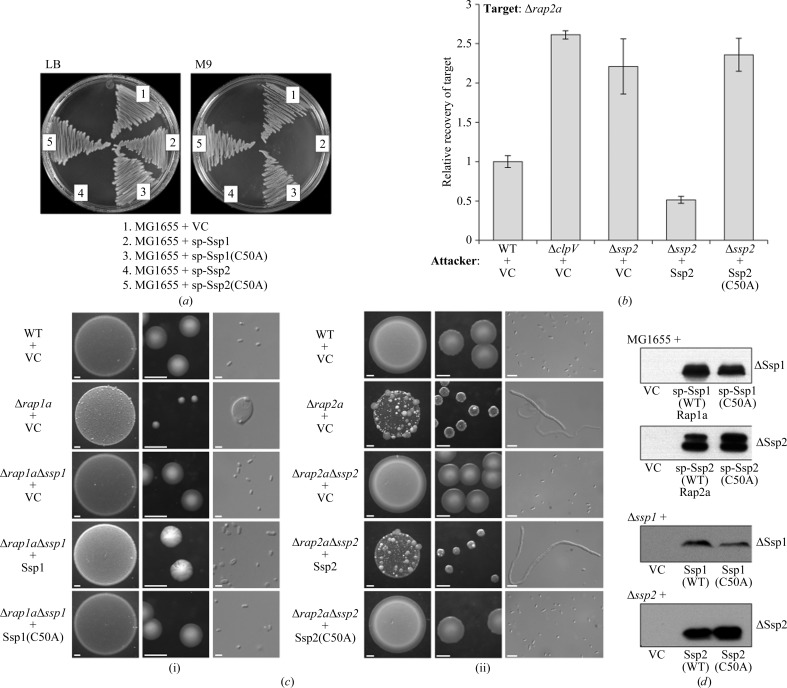
The Ssp1 and Ssp2 C50A mutants are inactive. (*a*) Growth of *E. coli* MG1655 transformed with plasmids expressing OmpA_sp_-Ssp1 (sp-Ssp1; pSC152), OmpA_sp_-Ssp1(C50A) [sp-Ssp1(C50A); pSC548], OmpA_sp_-Ssp2 (sp-Ssp2; pSC138) or OmpA_sp_-Ssp2(C50A) [sp-Ssp2(C50A); pSC549] from an arabinose-inducible promoter, or with the empty vector (VC, pBAD18-Kn), on LB or M9 medium containing 0.2% arabinose. (*b*) Recovery of a sensitive Δ*rap2a* mutant as the target strain following co-culture with the different attacking strains indicated, expressed relative to recovery of target when co-cultured with the wild-type strain. Attacking strains are wild-type *S. marcescens* Db10 (WT), mutant lacking ClpV (Δ*clpV*), mutant lacking Ssp2 (Δ*ssp2*), each carrying either the vector control plasmid (+VC; pSUPROM) or a plasmid expressing wild-type Ssp2 (+Ssp2; pSC541) or the C50A mutant of Ssp2 [+Ssp2(C50A); pSC1230]. (*c*) Phenotypes of wild-type *S. marcescens* Db10 and selected single and double mutants carrying plasmids expressing variants of Ssp1 or Ssp2 following growth on solid medium. For each strain, representative images of the morphology of a culture spot (left; scale bar 1 mm), single colonies (middle; scale bar 1 mm) and individual cells [right; scale bar 2 µm in (i) or 5 µm in (ii)] are shown. Plasmids direct the expression of wild-type Ssp1 (+Ssp1; pSC539), the C50A mutant of Ssp1 [+Ssp1(C50A); pSC1229], wild-type Ssp2 (+Ssp2; pSC541), the C50A mutant of Ssp2 [+Ssp2(C50A); pSC1230] or represent the vector control (+VC, pSUPROM). Growth was for 48 h on MM (i) or 24 h on LB (ii). (*d*) Immunoblot analysis of levels of Ssp1 or Ssp2 proteins from *E. coli* MG1655 (top two panels) or *S. marcescens* (bottom two panels) grown on solid medium. Strains and plasmids are as in (*a*)–(*c*), except that wild-type OmpA_sp_-Ssp1 and OmpA_sp_-Ssp2 were co-expressed with their cognate immunity proteins to allow the strains to grow [sp-Ssp1(WT)Rap1a; pSC160 or sp-Ssp2(WT)Rap2a; pSC144].

Established functional assays were utilized to examine the effect of mutating the catalytic cysteine (Cys50) of Ssp1 and Ssp2. Heterologous expression targeting Ssp1 and Ssp2 to the periplasm of *E. coli* MG1655 prevents growth on M9 minimal medium and, in the case of Ssp2, also on LB medium. As shown in Fig. 2 ▶(*a*), Ssp1-C50A and Ssp2-C50A were no longer toxic to *E. coli*. In *S. marcescens* itself, a mutant lacking the immunity protein, Δ*rap2a*, is susceptible to self-killing by Ssp2 injected by the wild-type strain. This is shown in two ways. Firstly, co-culture of a wild-type attacker strain with a Δ*rap2a* target strain results in the death of the latter. When the attacker lacks a functional T6SS (Δ*clpV*) or Ssp2 (Δ*ssp2* mutant), increased recovery of the target strain is observed. Complementation of the Δ*ssp2* mutant attacker by expression of wild-type Ssp2 from a plasmid restores the killing activity, with the recovery of the target reduced below the levels observed with the wild-type attacker. However, the expression of Ssp2-C50A was unable to complement the Δ*ssp2* mutant and restore the killing activity (Fig. 2 ▶ *b*). Secondly, a single Δ*rap2a* mutant exhibits fitness and morphological defects when grown on solid medium and these defects are alleviated when *ssp2* is also deleted in a Δ*rap2a*Δ*ssp2* mutant. Plasmid-mediated expression of Ssp2 in the Δ*rap2a*Δ*ssp2* mutant re-induced the fitness and morphological defects caused by self-killing; however, expression of the Ssp2-C50A mutant had no negative effect (Fig. 2 ▶ *c*). Similarly, expression of Ssp1, but not Ssp1-C50A, in a Δ*rap1a*Δ*ssp1* mutant induced mild morphological defects (Fig. 2 ▶ *c*). In each case, an equivalent level of stable expression of Ssp1-C50A and Ssp2-C50A compared with wild-type Ssp1 and Ssp2 was confirmed by immunoblotting (Fig. 2 ▶ *d*).

### 3.2. Catalytic inactivation of Ssp1 and Ssp2 does not affect their secretion or immunity protein binding   

It was necessary to confirm that the above observations were not affected by any influence of the catalytic C50A mutation on immunity protein binding or secretion of the effectors. Each of the Ssp C50A mutants was mixed with an equimolar amount of the cognate Rap protein, followed by size-exclusion chromatography analysis to monitor complex formation. In each case, a single species of apparent molecular weight ∼55 kDa was observed (Supplementary Fig. S1*a* 1). This behaviour exactly matches that of the wild-type proteins (English *et al.*, 2012 ▶) and indicates that complexes consisting of two Ssp and two Rap polypeptides are formed. It was also demonstrated that the Ssp2-C50A mutant protein was secreted by the T6SS of *S. marcescens* as efficiently as the wild-type protein (Supplementary Fig. S1*b*). We conclude that mutation of the catalytic cysteine in Ssp1 and Ssp2 abolishes their bacteriolytic effect because the dl-endopeptidase activity is compromised. Furthermore, we can conclude that the Ssp–Rap interaction does not depend on the catalytic cysteine and that recognition of the Ssp effectors by the T6SS machinery is also independent of their enzymatic activity.

### 3.3. Structure of Ssp1   

To gain a detailed insight into the structure and activity of the peptidoglycan endopeptidase effector Ssp1, we applied crystallographic methods. Isomorphous crystal structures of Ssp1 (at 1.85 Å resolution) and the active-site Ssp1-C50A mutant (at 2.2 Å resolution) reveal the overall enzyme structure and details of the active site. The protein is a monomer in solution but crystallized with two molecules in the asymmetric unit, each consisting of residues 1–163. The root-mean-square deviations (r.m.s.d.s) between least-squares superimposed C^α^ atoms gives an average of 0.3 Å when comparing the four polypeptides in the crystal structures. The copies in the asymmetric unit are therefore judged to be essentially identical and neither mutagenesis of the catalytic Cys50 nor crystal lattice packing interactions induces any major structural changes.

**Figure 3:**
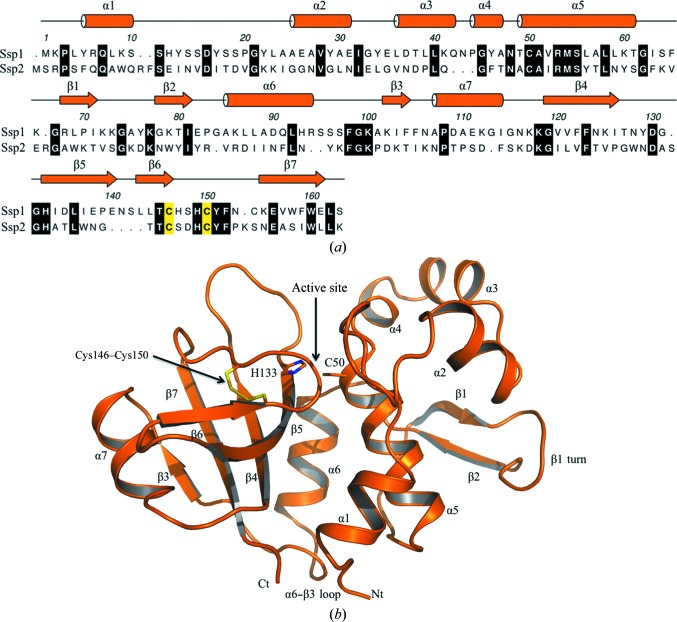
Primary, secondary and tertiary structure of Ssp1. (*a*) The amino-acid sequence of Ssp1 with assigned elements of secondary structure. Helices are depicted as cylinders and β-strands as arrows. An alignment of Ssp1 and Ssp2 is also shown with strictly conserved residues shown on a black background. Residues involved in disulfide-bond formation are coloured yellow. (*b*) Cartoon representation of Ssp1 with secondary-structure elements labelled. The disulfide bond is shown in stick representation with C-atom positions in yellow and S-atom positions in gold. His133 and Cys50 (sticks) mark the active site.

The primary, secondary and tertiary structure of Ssp1 (Fig. 3 ▶) classifies it into the NlpC/P60 cysteine peptidase superfamily (Anantharaman & Aravind, 2003 ▶). The overall dimensions of the bilobal structure are about 35 × 40 × 50 Å. An N-terminal subdomain comprises residues 1–95 and is formed by six α-helices (α1–α6) and, on the surface of the protein, a β-hairpin-like loop (β1, turn, β2). A short peptide segment links α6 to β3, which represents the start of the C-­terminal subdomain. This subdomain is dominated by a four-stranded antiparallel β-sheet with order β3–β7–β4–β5, which is flanked on one side by α7 and β6 and on the other by α6. A disulfide bond is observed between Cys146 at the C-­terminal end of β6 and Cys150 located on the loop linking β6 to β7. A cleft, the substrate-binding site, is created between the subdomains by residues in the α1–α2 and α4–α5 links, the C-terminal segment of β6 and the loop leading to β7. The disulfide linkage appears important to create one side of the active site and perhaps also contributes to the stability of the C-­terminal subdomain fold.

**Figure 4:**
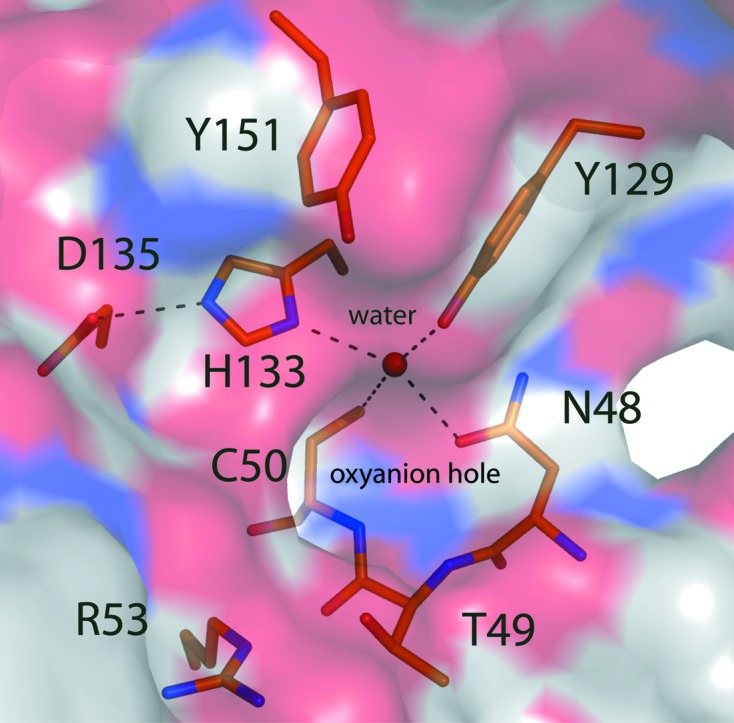
The Ssp1 catalytic centre. The protein is shown as a semi-transparent van der Waals surface coloured according to atom type (N, blue; O, red; S, yellow; C, white). A water (blue sphere) participates in four hydrogen-bonding interactions with Asn48, Cys50, Tyr129 and His133. Hydrogen bonds are shown as dashed lines and selected residues are presented as sticks with C-atom positions coloured orange, N blue, O red and S yellow.

The family 4 amidase effectors hydrolyze the amide bond between d-Glu and *m*A_2_pm. They are similar to CHAP (cysteine, histidine-dependent amidohydrolases/peptidases) family members, a subset of the NlpC/P60 superfamily with a strictly conserved cysteine–histidine catalytic dyad (Fyfe *et al.*, 2008 ▶). In the case of Ssp1, Cys50 is located in the N-terminal subdomain and His133 in the C-terminal subdomain (Fig. 3 ▶ *b*). Cys50 donates a hydrogen bond to a water molecule, which in turn interacts with His133, Asn48 and Tyr129 (Fig. 4 ▶). Asp135 accepts a hydrogen bond from His133 and appears to complete a catalytic triad reminiscent of that observed in typical cysteine peptidases. The mechanism of such cysteine-dependent proteases, especially papain, is well documented (Alphey & Hunter, 2006 ▶ and references therein). The Cys–His catalytic dyad forms a thiolate–imidazolium pair that is oriented by a hydrogen bond between the histidine and an acidic residue. In this case the residues are Cys50, His133 and Asp135. The generation of a thiolate Cys50 following proton abstraction by His133 would support nucleophilic attack at the γ-d-glutamyl-*m*A_2_pm amide bond to form an acyl thioester. Hydrolysis of the thioester, exploiting an activated water molecule, then releases the products. The main-chain amides of the catalytic cysteine and Thr49 create an oxyanion hole that may support thiolate attack on the amide linkage by stabilizing the tetrahedral intermediate formed prior to the formation of an acyl-enzyme complex. In this there are striking parallels to the reaction catalyzed by nicotinamidase (Fyfe *et al.*, 2009 ▶).

Ssp1 shares about 20% sequence identity with Ssp2, but we do not have a structure of Ssp2 for comparative purposes to address the observation of distinct substrate specificities. However, the closest structural relative of Ssp1 is the bacteriolytic effector Tae4 from *E. cloacae* and *S. typhimurium* (PDB entries 4hfk and 4hff, respectively; Zhang *et al.*, 2013 ▶), with *Z*-­scores of 17 and 16 and r.m.s.d.s of 2.2 and 2.1 Å for the least-squares fit of 138 and 137 C^α^ positions, respectively. Ssp1 shares about 15% sequence identity with these proteins but, despite such a low sequence similarity, the r.m.s.d. values and overlay confirm that these structures share the same fold (Supplementary Fig. S2). On the basis that Ssp2 shares about 50% sequence identity with Tae4 and the same substrate specificity, we suggest that it provides a suitable surrogate structure for comparative purposes.

An overlay of the Ssp1 and Tae4 structures confirms similarities in the protein fold and the relative positions of residues linked to catalytic activity. However, the overlay also indicates noteworthy differences (Supplementary Figs. S2 and S3). The α1–α2 loop in Ssp1, consisting of about 15 amino acids, lines one side of the active site then extends away from the catalytic core. Adjacent to this loop is a turn carrying the Cys146 and Cys150 disulfide. The turn and the polypeptide that follows, which lead directly into β7, line the active site. In *Ec*Tae4, the disulfide is conserved (Cys137 and Cys141), as is the structure just after the turn. An eight-residue segment extends from the turn and curls over to narrow the active-site cleft. In *S*TTae4 the conserved residues Cys135 and Cys139 are in a reduced form, for reasons that are not made clear, and the polypeptide chain at the periphery of the active site is flexible and dis­ordered, as shown by inflated thermal parameters and a lack of electron density. Alignment of the Ssp1 and Ssp2 amino-acid sequences (Fig. 3 ▶ *a*) indicates that there is a truncation of four residues in the region of the disulfide-containing loop and this may reduce the size of the active-site cleft of Ssp2 compared with Ssp1. The alignment also suggests that Asp135 of Ssp1, assigned as a component of the catalytic triad, is not conserved and corresponds to Thr133 in Ssp2. In *S*TTae4 and *Ec*TAE4 a threonine is also present at this position (Supplementary Fig. S4). A superposition of Ssp1 and *Ec*TAE4 indicates a difference of Ser148 replaced by Asp139, with the carboxylic acid side chain positioned to fulfil the role of Asp135 of Ssp1 in the catalytic triad (Supplementary Fig. S3). The aspartate is conserved in Ssp2, *S*TTae4 and *Ec*TAE4 (Supplementary Fig. S4).

The peptidoglycan hydrolase assay data indicate that Ssp2 preferentially targets the acceptor component of the peptidoglycan tetratetra cross-link rather than donor part and in this it is similar to Tae4 (Russell *et al.*, 2012 ▶). Ssp1 cleaves the acceptor and donor stem of cross-­linked and non-cross-linked peptidoglycan. The differences evident from structural comparisons suggest that a distinctive and more open substrate-binding surface in Ssp1 compared with those observed in Tae4 structures or implied in Ssp2 might explain the promiscuous endopeptidase activity of Ssp1. Structures of complexes with the appropriate ligands would be required to further investigate this point.

### 3.4. Structure of Rap1a   

**Figure 5:**
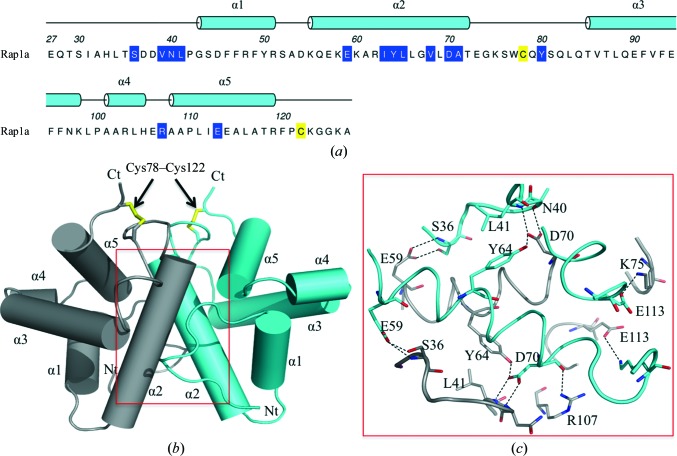
Primary, secondary and tertiary structure of Rap1a. (*a*) The sequence of mature Rap1a. The 26-residue N-­terminal signal sequence is not shown. These residues represent the cleaved N-terminal signal sequence that was omitted from the analysis. The assigned α-helical secondary structure is shown and the helices are numbered. Residues involved in disulfide-bond formation are coloured yellow and residues that contribute to the dimer interface are shown on a blue background. (*b*) Cartoon representation of the Rap1a dimer with labelled helices; Nt and Ct mark the N- and C-terminal positions. The disulfides formed between Cys78 and Cys122 are shown as yellow sticks. (*c*) The residues and hydrogen bonds (dashed lines) at the dimer interface. C atoms are shown in grey and cyan to distinguish the subunits.

Next, we sought to reveal molecular details of the immunity proteins Rap1a and Rap2a, which neutralize Ssp1 and Ssp2 toxicity, respectively (English *et al.*, 2012 ▶). The structure of Rap1a was determined to about 2.0 Å resolution (Table 1 ▶) and the amino-acid sequence with assigned secondary structure is depicted in Fig. 5 ▶(*a*). A search for structural orthologues failed to identify anything of relevance in the PDB. We therefore conclude that the Rap1a fold has not been observed previously, making it a unique member of the T6SS immunity family of proteins. Several other candidate immunity proteins identified in other organisms share significant sequence identity with Rap1a (see below). Therefore, we propose that this group of proteins be referred to as the ‘Tai4a’ immunity proteins, to reflect the fact that whilst they are immunity proteins to Tae4 effectors, they are distinct from the main family of Tai4 proteins, which includes Rap1b, Rap2a and Rap2b.

The Rap1a subunit displays a compact globular structure constructed from five α-helices that assemble to form the highly stable symmetric dimer that constitutes the asymmetric unit (Fig. 5 ▶). This is consistent with the size-exclusion chromatography data, which identified that only a dimer was observed in solution (Supplementary Fig. S1). The NCS is highly conserved, with an r.m.s.d. of 0.6 Å for a least-squares overlay of 96 C^α^ positions. A disulfide bond is formed between Cys78 (in the α2–α3 loop) and Cys122 in the C-terminal region. This interaction appears to be crucial to stabilizing the subunit fold since it helps position α2, α3 and α5 close to each other and these segments of secondary structure provide the side chains that form the hydrophobic core of the subunit. During initial recombinant expression tests it was noted that soluble protein was only produced in *E. coli* Rosetta-gami (DE3) cells, a strain that promotes the formation of disulfide bonds in the cytoplasm and so mimics what might occur in the oxidative environment of the periplasm. This suggests that the covalent bond is necessary for correct folding to occur and for stability of the Rap1a fold and dimeric quaternary structure.

Interactions involving residues in α2 make the major contribution to dimer formation. Each subunit contributes a surface area of 1130 Å^2^ to the dimer interface, which is 20% of the solvent-accessible surface area (ASA) of the subunit (5700 Å^2^). Such a percentage of surface area is indicative of a stable association (Krissinel & Henrick, 2007 ▶). Eight residues from each subunit form a network of hydrogen bonds using both main-chain and side-chain functional groups (Ser36, Asn40, Leu41, Glu59, Tyr64, Asp70, Lys75, Arg107 and Glu113, Fig. 5 ▶ *c*). There are also indirect hydrogen-bonding interactions *via* well ordered water molecules that link a number of side chains and main chains. Hydrophobic interactions that contribute to the stability of the dimer mainly involve the aliphatic side chains of Val39, Ile63, Leu66, Val68 and Ala71, but also Tyr80.

### 3.5. Structure of Rap2a   

Rap2a, like Rap1a, is predicted to be localized in the periplasm and could only be produced in soluble recombinant form using the *E. coli* Rosetta-gami (DE3) strain. It is also a stable dimer in solution as shown by size-exclusion chromatography (English *et al.*, 2012 ▶). The structure was determined at 1.9 Å resolution with four molecules, arranged as two dimers, in the asymmetric unit. These four molecules, sub­units *A*–*B* and *C*–*D*, are similar overall, with r.m.s.d.s between superimposed C^α^ atoms ranging from 0.6 Å (subunits *A* and *B*, residues Thr27–Gln123) to 0.7 Å (subunits *C*, residues 27–127, and *D*, residues 27–126). Minor deviations from NCS are observed in a five-residue loop between α2 and α3 (Gly67–Leu71) and indicate some conformational freedom in this part of the molecule. The ASA of a Rap2a subunit averages at approximately 6110 Å^2^; the range is from 6140 Å^2^ for subunit *C* to 6090 Å^2^ for subunit *D*. Dimer formation occludes an area that is approximately 20% of the ASA, which is indicative of a stable association (Krissinel & Henrick, 2007 ▶).

**Figure 6:**
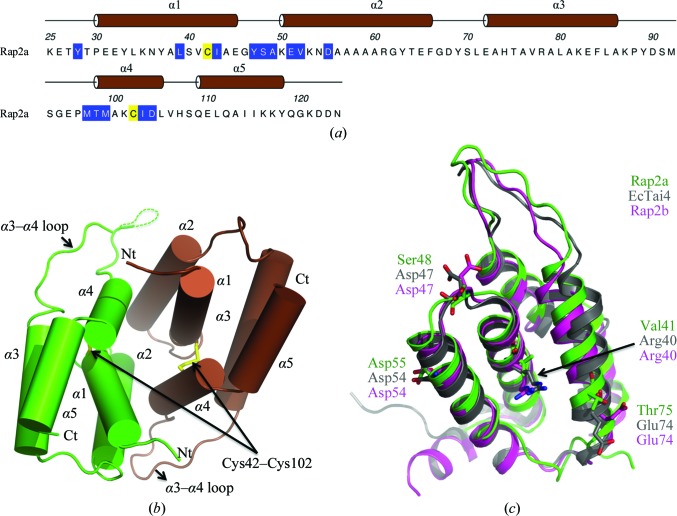
Primary, secondary and tertiary structure of Rap2a. (*a*) The amino-acid sequence with assigned secondary structure and helices numbered. Residues involved in disulfide-bond formation are coloured yellow and residues involved in subunit–subunit interactions are shown on a blue background. (*b*) Cartoon representation of the Rap1a dimer with subunits coloured brown and green. The disulfides and the N- and C-termini are labelled. (*c*) Cartoon representation of Rap2b (pink) and *Ec*Tai4 (grey) superimposed on Rap2a (green). Asp54 and Asp47 are involved in the hydrogen-bond network in the interface in *Ec*Tai4 and are absolutely conserved in Rap2b; in Rap2a, Asp47 is replaced by Ser48. Arg40 and Glu74 (replaced by Val41 and Thr75, respectively, in Rap2a) have been shown to play a major role in binding to *Ec*Tae4; both of these residues are also conserved in Rap2b but not in Rap2a.

The Rap2a subunit displays a compact globular structure of five α-helices with an extended loop linking α3 to α4 (Figs. 6 ▶ *a* and 6 ▶ *b*). A disulfide bond between Cys42 and Cys102 links α1 to α4 and interactions of other side-chain groups on these elements of secondary structure help to create the helical bundle fold and in particular to align α2. Together, α1, α2, the α2–α3 loop and α4 form the dimer interface, giving rise to a twofold NCS axis, and a combination of hydrogen-bonding, salt-bridge and van der Waals interactions serve to stabilize the association. Main-chain hydrogen-bonding contributions come from the amides of Ser48, Ala49, Met97, Thr98 and Met99 on both chains. Side-chain contributions come from the hydroxyl groups of Tyr28 and Tyr47 and the carboxylates of Glu51, Asp55 and Asp104. Several solvent-mediated contacts also serve to link functional groups on partner subunits (not shown). The side chains of Leu39, Ile43, Tyr47, Val52, Met99 and Ile103 are involved in van der Waals interactions with the partner subunit to stabilize the dimer.

Despite a low level of sequence conservation, the structural similarity of five T6SS immunity proteins (Rap1b, Rap2a, Rap2b, *Ec*Tai4 and *S*TTai4) indicates an orthologous subset. This structurally defined set is consistent with the designation of a ‘Tai4’ family of immunity proteins cognate to the Tae4 effectors (Russell *et al.*, 2012 ▶). Pairwise comparisons indicate a range of sequence identities from 15 to 35%, and matching between 93 and 124 C^α^ positions gives an r.m.s.d. range of 1.2–1.7 Å indicative of close structural similarity. The structural overlay is exemplified by superposition of Rap2a, Rap2b and *Ec*Tai4 (Fig. 6 ▶ *c*). Of note is the conservation of an extended or protruding loop structure which is highly variable in terms of amino-acid sequence (see Fig. 7 in English *et al.*, 2012 ▶). Parts of the *Ec*Tai4 subunit involved in interaction with an effector, identified by the positions of Val41 and Thr75 in Rap2a, are also well conserved in terms of overall structure (Fig. 6 ▶ *c*).

This subset of immunity proteins also display similar dimer structures and the Cys42–Cys102 disulfide bond in Rap2a is conserved in Rap1b and Rab2b. The covalent interaction appears to be important for the creation of the subunit fold and the stable quaternary structure. In the structures of *Ec*Tai4 and *S*TTai4 there are conserved cysteines that match the disulfide-forming residues in the Rap proteins; however, they are in a reduced form. In *S*TTai4, for example, the distance between the SG atoms of Cys48 and Cys108 is 3.6 Å and the electron density unambiguously defines reduced cysteine residues. This difference in the redox states may simply reflect distinct experimental conditions.

### 3.6. Inhibition of Ssp1 by Rap1a   

We next sought to delineate the molecular basis of how the *S. marcescens* Rap proteins neutralize their cognate Ssp amidase/endopeptidase effectors and what features engendered the exquisite specificity noted in the effector–resistance protein combinations. We previously proposed that the complexes exist as Ssp_2_–Rap_2_ heterotetramers based on biochemical analyses (English *et al.*, 2012 ▶), a conclusion that was subsequently confirmed by the structures of related Tae4–Tai4 complexes (Zhang *et al.*, 2013 ▶). The similarities of Ssp2 and Rap2a to Tae4 and Tai4 in terms of peptidoglycan hydrolase activity, sequence and structure suggested that the mode of effector inhibition is similar and this will be discussed below. In contrast, Ssp1 displays differences in activity and structure from Tae4 and is biologically distinct from Ssp2. In addition, the structure of Rap1a presents a new fold distinct from the Tai4 proteins; hence, it was important to elucidate the structure of an Ssp1–Rap1a complex.

**Figure 7:**
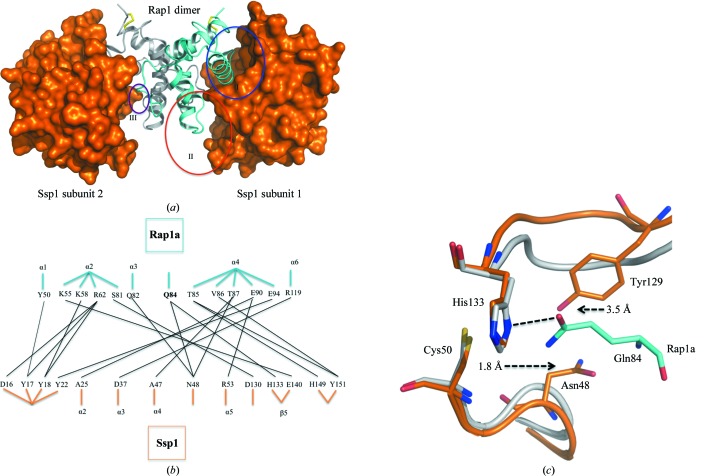
The Ssp1–Rap1a heterotetramer complex. (*a*) Ssp1 is shown as an orange van der Waals surface and the Rap1a subunits are shown as cyan and grey ribbons. The disulfide bonds in Rap1a are presented as yellow sticks. Blue, red and purple circles mark the three distinct areas (I–III) of interaction between the effector and immunity proteins. (*b*) Schematic diagram showing the hydrogen-bonding and salt-bridge interaction network involved in complex formation. Black lines mark interacting residues. Residue positions in the protein structures are given by labelling the appropriate secondary structure. The exceptions are Gln84 in Rap1a, which occurs just prior to α3 in this structure, and Asn48 and Asp130 in Ssp1, which are between α4 and α5 and at the C-terminal segment of β4, respectively. (*c*) Gln84 directly interacts with the catalytic His133 and blocks the active site. Grey, apo Ssp1; dark orange, Ssp1 from the Ssp1–Rap1a complex; cyan, Rap1a from the Ssp1–Rap1a complex. Broken arrows indicate the adjustments of Asn48 and Tyr129 when comparing free and complexed Ssp1.

Following purification using a co-expression strategy, we determined the structure of the Ssp1–Rap1a complex at about 2.0 Å resolution. There are two molecules in the asymmetric unit (one Ssp1 and one Rap1a) and a crystallographic twofold axis given by the symmetry operation *x* − *y*, −*y*, −*z* + 2/3 generates a heterotetramer (Fig. 7 ▶ *a*). The overall dimensions of this heteromeric assembly are about 90 × 50 × 50 Å and its molecular weight is approximately 60 kDa, consistent with that observed by size-exclusion chromatography during purification of the complex (Supplementary Fig. S1). The model contains residues 1–163 of Ssp1 and residues 30–123 of Rap1a. The solvent-accessible surface area of Ssp1 is 5840 Å^2^ and about 16% of this (960 Å^2^) is occluded when the complex with Rap1a is formed.

Rap1a interacts with Ssp1 using residues in α1, α3, α4, α5 and the α4–α5 loop (Fig. 7 ▶). In particular, the α4–α5 loop and α4 of Rap1a are directly positioned to block the Ssp1 active site. An extensive network of hydrogen bonds, van der Waals forces and water-mediated hydrogen bonds are present at the Ssp1–Rap1a interface. These interactions occur primarily in two areas (areas I and II in Fig. 7 ▶ *a*). A total of 13 Ssp1 residues (Asp16, Tyr17, Ser18, Tyr22, Ala25, Asp37, Ala47, Asn48, Arg53, Asp130, His133, His149 and Tyr151) and 12 Rap1a residues (Tyr50, Lys58, Arg62, Ser81, Gln82, Gln84, Thr85, Val86, Thr87, Glu90, Glu94 and Arg119) contribute to the network of interactions and the details are presented in a schematic form in Fig. 7 ▶(*b*). Gln84 of Rap1a forms a hydrogen bond to the N^δ^ atom of the catalytic His133 of Ssp1 and is a clear marker of the steric block provided by the immunity protein (Figs. 7 ▶ *b* and 7 ▶ *c*). Arg119, Arg66, Lys59, Glu94 and Glu94 of Rap1a form salt-bridge interactions with Asp37, Asp16, Glu140, Arg53 and Arg53 of Ssp1, respectively. Tyr50 in α1 of Rap1a interacts with Tyr17 from Ssp1 through a hydrogen bond and π-stacking. A small interface (100 Å^2^) is created where Asp36 from Rap1a and His149 from Ssp1 interact (area III in Fig. 7 ▶).

An overlay (not shown) of the two Rap1a structures, alone and in complex with Ssp1, gives an r.m.s.d. of 0.4 Å for 94 C^α^ atoms, indicating a high similarity in overall structure with no major conformational differences. Rap1a therefore appears to be a highly stable pre-formed binding partner for Ssp1 when in the correct redox state. There is, however, a localized effect following complex formation. In Rap1a, the segment linking α2 and α3, residues 74–84, shows high average *B* factors (temperature factors; 76.4 Å^2^) compared with the overall average *B* factor (31.5 Å^2^) and relatively diffuse electron density. This region contributes to Ssp1–Rap1a complex formation and becomes well ordered, with an average *B* factor of 15.6 Å^2^ compared with the overall *B* factor of 22.6 Å^2^ for the Rap1a component of the complex. The overall structure of Ssp1 is also well retained between the free and complexed states, with an r.m.s.d. of 0.8 Å for the least-squares fit of 162 C^α^ atoms. There are localized adjustments which appear to support complex formation. In the α1–α2 loop of Ssp1, Tyr17 is relocated by about 7 Å to interact with Tyr50 and Arg62 of Rap1a. In addition, Asn48 and Tyr129 of Ssp1 move 1.8 and 3.5 Å outwards and away from the catalytic centre (Fig. 7 ▶ *c*).

The Tae4–Tai4 complex from *E. cloacae* also forms a heterotetrameric assembly, with the Tai4 dimer forming the central segment and a Tae4 effector at either end (Zhang *et al.*, 2013 ▶). The complex is stabilized by extensive noncovalent interactions formed between the effector and both Tai4 subunits. The protruding loop (Fig. 6 ▶ *c*) of one Tai4 subunit binds in the active site of the effector and helices α2 and α3 of the other subunit are positioned to interact with residues in the N-terminal subdomain. This Tae4–Tai4 complex is likely to be a good model for the Ssp2–Rap2a complex. The close structural relationship, in terms of fold, for both the effector and the immunity proteins (see, for example, Fig. 6 ▶) would suggest that Rap2a would interact with Ssp2 in a similar fashion and that the selectivity of immunity proteins with the Rap2a fold for or against other enzymes would be determined by variation in the side chains. Such differences are likely to involve residues in the loops linking α2 to α3 and α3 to α4 in the immunity proteins, which interact with the α3–α4 section, the β4–β5 loop and the N-terminal section of β7 in some Tae4 proteins. These parts of the proteins are poorly conserved in terms of sequence identity (Supplementary Figs. S4 and S5).

However, the complex assembly that leads to inhibition is completely distinct between Ssp1 and the other Tae4 systems. Rap1a, which has a completely different fold to the conventional Tai4 proteins, does not possess a protruding loop, but in the complex a helix is positioned to block the effector active site and the interactions that stabilize the complex involve residues in the C-terminal subdomain of the effector rather than the N-terminal subdomain (Supplementary Fig. S6).

### 3.7. Co-evolved diversity within the Tae4/Ssp and Tai4/Rap families   

Finally, we considered the question of why *S. marcescens* has two Tae4 homologues (Ssp1 and Ssp2) and four Rap-family proteins rather than just one amidase effector–immunity combination. Ssp1 and Ssp2 are not redundant since they elicit different biological consequences in a target cell. For example, as shown here and previously (Fig. 2 ▶; English *et al.*, 2012 ▶), an immunity mutant suffering Ssp2-mediated killing is highly unfit and displays striking cell filamentation, whereas an immunity mutant suffering Ssp1-mediated killing is less disabled and shows round enlarged cells. The difference between the Ssp1-mediated and Ssp2-mediated effects may be related to the apparent modulation of substrate preference observed in the *in vitro* enzyme assays, perhaps reflecting differences in the extent or pattern of cell-wall damage (Fig. 1 ▶). This study has unexpectedly revealed that the cognate immunity protein for Ssp1, Rap1a, has a distinct structure compared with the other Rap and Tai4 proteins studied to date (Fig. 5 ▶).

**Figure 8:**
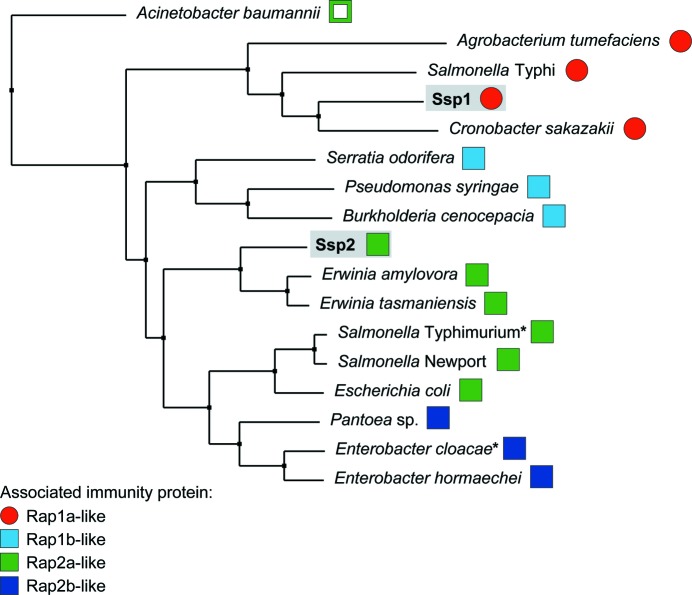
Subgroups of Tae4 family proteins and their associated immunity proteins. Neighbour-joining tree calculated from a multiple sequence alignment of Ssp1, Ssp2 and other Tae4 homologues from different bacterial species (a full alignment is shown in Supplementary Fig. S6). For each Tae4 protein, the adjacently encoded candidate immunity protein was identified by genomic analysis and the *S. marcescens* Rap protein to which it was most closely related was determined. Tae4 homologues with associated immunity proteins similar to Rap1a (Tai4a) are shown by red circles, whereas those with immunity proteins of the Rap1b/Rap2a/Rap2b (Tai4) type are shown by squares (in light blue, green or dark blue for most similarity to Rap1, Rap2a or Rap2b, respectively). Apart from Ssp1 and Ssp2 from *S. marcescens*, the Tae4 homologues are labelled by organism and their identities are as follows (UniProt or genomic identifiers): *Acinetobacter baumannii*, B0VVE3_ACIBS; *Agrobacterium tumefaciens*, Atu4347; *Burkholderia cenocepacia*, Bcen_4030; *Cronobacter sakazakii*, ESA_03935; *Enterobacter cloacae*, ECL_01542; *Entero­bacter hormaechei*, F5RYK9_9ENTR; *Erwinia amylovora*, EAMY_3018; *Erwinia tasmaniensis*, ETA_06210; *Escherichia coli*, ECEG_03250; *Pantoea* sp., S7A_11480; *Pseudomonas syringae*, Psyr_4040; *Salmonella enterica* serovar Newport, SNSL254_A0303; *S. enterica* serovar Typhimurium, STM0277; *S. enterica* serovar Typhi, STY0307; *Serratia odorifera*, D4E4R6_SEROD. Asterisks indicate the Tae4–Tai4 homologues for which structures have previously been reported (Zhang *et al.*, 2013 ▶) and the open square indicates that this candidate immunity protein showed only weak similarity to Rap2a. Details of the Tae4 homologues and associated immunity proteins are given in Supplementary Table S1.

A comparison of the amino-acid sequences of Ssp1, Ssp2 and Tae4 homologues showed that Ssp1 and several other Tae4 homologues form a distinct grouping (Fig. 8 ▶). This is consistent with several structural differences being observed in Ssp1 compared with *Ec*Tae4 (Supplementary Fig. S2). When the adjacently encoded known or putative immunity protein was identified for all of these effectors and compared with the four *S. marcescens* Rap proteins, a pattern became evident. The Ssp1-like proteins all co-occur with immunity proteins of the Rap1a type. The other Tae4 homologues are less separated from each other, but there is a clustering of Tae4 proteins sharing related immunity proteins. So, for example, the three Tae4 proteins whose immunity proteins are most closely related to Rap1b all cluster together (Fig. 8 ▶) and the Tae4 homologues most similar to Ssp2 all have associated Rap2a-like immunity proteins (Fig. 8 ▶). Hence, effector and immunity proteins appear to have co-evolved within the Tae4 and Tai4 family. In particular, the Ssp1-like proteins appear to form a subgroup distinct enough to utilize a structurally unrelated immunity protein (‘Tai4a’), exemplified by Rap1a. The reason for this divergence is unclear, although it is consistent with the distinct biological phenotypes associated with the two effectors. Having both Ssp1 and Ssp2 is likely to confer an evolutionary advantage on the secreting organism, perhaps with each being more efficient against different target species or under different growth conditions than the other. Additionally, having Ssp1 may allow attack on a close relative with Ssp2/Rap2a and *vice versa*, maximizing the ability to distinguish ‘self’ from competitors.

This analysis strongly suggests that Rap1b and Rap2b are not ‘inactive’ immunity proteins; rather, they are likely to provide protection against incoming Tae4 proteins from other T6SS-elaborating bacterial species. Rap1b is most closely related to immunity proteins associated with Tae4 proteins (Fig. 8 ▶) and thus would be expected to bind an effector of this type and not Ssp1- or Ssp2-like Tae4 proteins. Similarly, Rap2b may neutralize Tae4 proteins related to *Ec*Tae4 (Fig. 8 ▶). This idea would also predict that neither Rap1b nor Rap2b would bind Ssp1 or Ssp2, and indeed we have shown previously that they do not (English *et al.*, 2012 ▶). Other bacteria can also be observed to possess ‘extra’ Tai4 proteins compared with their Tae4 complement (this study and Russell *et al.*, 2012 ▶). For example, *Cronobacter sakazakii* has ‘orphan’ Rap1a, Rap2a-like and Rap2b-like proteins (ESA_03939, ESA_03933 and ESA_03932) in addition to its Ssp1–Rap1a pairing. Thus, one could envisage an ‘arms race’ in which attackers can gain an advantage by acquiring a Tae4 protein of a different subgroup whilst targets can counter by acquiring an immunity protein of the matching type.

## 4. Concluding remarks   

Our structural and activity data, and comparisons with related systems, reveal that the T6SS family 4 endopeptidase effectors are likely to share the same enzyme mechanism but fall into two functional categories in terms of overall structure and substrate processing. One displays narrow specificity and one is more promiscuous. Some of the immunity proteins encoded within the T6SS gene clusters, those cognate to Tae4/family 4 effectors, can be placed in two protein-fold families which we suggest be termed (i) Tai4, as described previously and including Rap2a, *Ec*Tai4 and *S*TTai4, and (ii) Tai4a, including Rap1a and described for the first time here. Although distinct in terms of structure, the two families are built upon similar underlying principles, namely stable dimeric small α-helical bundles. These T6SS-associated immunity proteins and indeed the effectors in *S. marcescens* appear to rely on the formation of disulfide linkages for folding and activity.

Although the mode of effector inhibition is conserved in the two families, namely a steric block of the active site within a heterotetrameric complex, our study reveals that a very different structure can be used to accomplish effector neutralization. The inhibitory action of immunity proteins is therefore highly specific for effector proteins, even though some effectors have closely related enzyme activities. The type VI-associated endopeptidase effectors are highly basic proteins, as exemplified by Ssp1 and Ssp2, with predicted pI values of 9.1 and 9.3, respectively. These enzymes act on peptidoglycan and process a substrate in the vicinity of two acidic carboxylates, the d-Glu residues. The immunity proteins are acidic; the pI values for the four Rap proteins fall in the range 5.1–6.3 after omitting the signal peptides. Such complementarity of charge contributes to the high-affinity interactions that support complex formation, despite distinctive structures and variation in sequence, and may generate long-range electrostatic attraction to assist correct binding.

The exact mechanism by which the effector proteins are secreted using the T6SS is not yet known. One model suggests a needle comprising a channel formed by haemolysin-coregulated protein (Hcp) oligomers with an internal diameter reported as about 40 Å (Mougous *et al.*, 2006 ▶). How this value was estimated is not detailed. We would suggest that from measurements across the pore and taking van der Waals radii into consideration, 40 Å might be a generous estimate of the pore size. We analyzed the globular dimensions of the family 4 endopeptidase effectors and the largest, by a small margin, is Ssp1, with approximate dimensions of 35 × 40 × 50 Å, taking into consideration the van der Waals radii. Such effector proteins, when folded, are therefore comparable in size with or smaller than the pore of the Hcp oligomer. Some effectors may be translocated out of the cell in an intact folded and functional state (Benz *et al.*, 2012 ▶) and some may be only partially folded when they are the substrate for the T6SS (Chou *et al.*, 2012 ▶). It is possible that Ssp1, Ssp2 and related effectors may utilize disulfide bonds to ‘lock’ their final structure once translocated away from the reducing environment of the cytoplasm, either in the extracellular environment or within the periplasm of a target cell. In this sense, the T6SS may have evolved to exploit redox status in the periplasm both for arming effector warheads when engaged in attack or for generating a protective armour when in defensive mode.

## Supplementary Material

PDB reference: Ssp1, 4bi3

PDB reference: Ssp1-C50A, 4bi4

PDB reference: Ssp1–Rap1a, 4bi8

PDB reference: Rap1a, 3zfi

PDB reference: Rap2a, 3zib

Supporting information file. DOI: 10.1107/S0907444913022725/be5242sup1.pdf

## Figures and Tables

**Table 1:** Crystallographic statistics Values in parentheses are for the highest resolution shell.

Structure/PDB code	Ssp1/4bi3	Ssp1-C50A/4bi4	Rap1a/3zfi	Rap2a/3zib	Ssp1Rap1a/4bi8
Space group	*P*2_1_2_1_2_1_	*P*2_1_2_1_2_1_	*C*222_1_	*P*2_1_	*P*322_1_
Wavelength ()	1.5418	1.5418	0.97950	0.96110	1.5418
Unit-cell parameters					
*a* ()	56.83	56.79	82.65	39.65	68.47
*b* ()	65.26	64.59	93.00	81.35	68.47
*c* ()	97.50	97.87	51.26	58.45	92.52
()				91.54	90.00
Resolution range ()	48.751.85 (1.951.85)	48.932.21 (2.262.21)	46.501.98 (2.091.98)	40.671.90 (2.001.90)	49.922.00 (2.052.00)
No. of reflections	503341 (41592)	139598 (9390)	63106 (8849)	214423 (31351)	493039 (32290)
Unique reflections	31504 (4357)	18726 (1331)	14089 (2003)	29067 (4190)	17462 (1279)
Completeness (%)	99.4 (100.0)	99.9 (98.5)	99.9 (100.0)	99.4 (98.9)	99.9 (99.1)
*R*_merge_ † (%)	7.4 (23.9)	7.5 (26.3)	6.6 (48.6)	9.7 (51.6)	10.9 (58.7)
Multiplicity	16.0 (9.5)	7.5 (7.1)	4.5 (4.4)	7.4 (7.5)	28.2 (25.2)
*I*/(*I*)	33.7 (10.2)	23.0 (8.1)	13.4 (2.7)	15.3 (4.0)	40.9 (11.4)
Wilson *B* (^2^)	10.7	18.1	31.5	21.7	18.8
*R*_work_ ‡/*R*_free_ § (%)	20.4/24.0	19.2/24.6	19.2/23.4	18.3/23.4	17.9/22.7
No. of residues	326	326	185	377	256
No. of waters	300	372	57	137	192
No. of ligands	3 K^+^, 5 SO_4_^2^	1 glycerol			
DPI¶ ()	0.13	0.21	0.15	0.15	0.16
R.m.s.d. bond lengths†† ()	0.006	0.008	0.018	0.019	0.006
R.m.s.d. angles†† ()	0.967	1.301	1.925	1.745	1.020
Average *B* factors (^2^)					
Chain *A*	12.7	17.7	34.3	19.7	15.6
Chain *B*	11.2	17.9	35.2	23.0	22.6
Chain *C*				22.4	
Chain *D*				18.9	
Waters	15.2	25.1	34.8	30.6	27.0
K^+^	22.0				
SO_4_^2^	12.5				
Glycerol		35.9			
Ramachandran plot analysis					
Favoured regions	97.1	97.0	97.0	99.0	96.4
Allowed regions	2.9	3.0	3.0	1.0	3.2
Outliers	0	0	0	0	0.4 [Lys125]

† *R*_merge_ = 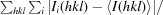 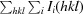, where *I_i_*(*hkl*) is the intensity of the *i*th measurement of reflection *hkl* and *I*(*hkl*) is the mean value of* I_i_*(*hkl*) for all *i* measurements.

‡ *R*_work_ = 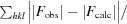 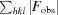, where *F*_obs_ is the observed structure factor and *F*_calc_ is the calculated structure factor.

§ *R*_free_ is the same as *R*_cryst_ except calculated with a subset (5%) of data that were excluded from the refinement calculations.

¶ Diffraction-component precision index (Cruickshank, 1999 ▶).

†† Engh Huber (1991 ▶).

## Footnotes

1 Supplementary material has been deposited in the IUCr electronic archive (Reference: BE5242). Services for accessing this material are described at the back of the journal.
