# Supplementary material for: Structural basis for type VI secreted peptidoglycan dl-endopeptidase function, specificity and neutralization in Serratia marcescens
Source: Acta Crystallogr D Biol Crystallogr. 2013 Nov 19;69(Pt 12):2468–82. doi: 10.1107/S0907444913022725 (PMC3852654; doi:10.1107/S0907444913022725)
Supplement: Supplementary file 1 [file d-69-02468-sup1.pdf]

Supplementary Material

Structural basis for Type VI secretion peptidoglycan DL-endopeptidase function, specificity and neutralization in *Serratia marcescens*.

Velupillai Srikannathasan, Grant English, Nhat Khai Bui, Katharina Trunk, Patrick E. F. O'Rourke, Vincenzo A. Rao, Waldemar Vollmer, Sarah J. Coulthurst* and William N. Hunter*

Supplementary Table S1. Ssp/Tae4 family proteins and corresponding adjacently-encoded immunity proteins.

Organism	Ssp/Tae4 protein		Adjacent Immunity Protein		
	UNIPROT identifier or name	Genomic identifier	UNIPROT identifier or name	Genomic identifier	Closest Rap
Agrobacterium tumefaciens C58	Q7CUP8_AGRT5	Atu4347	A9CGG9_AGRT5	Atu4346	Rap1a (SMA2260)
Burkholderia cenocepacia AU1054	Q1BN86_BURCA	Bcen_4030	Q1BN87_BURCA	Bcen_4029	Rap1b (SMA2262)
Cronobacter sakazakii ATCC BAA-894	A7MQ14_CROS8	ESA_03935	A7MQ15_CROS8[1]	ESA_03936	Rap1a (SMA2260)
Enterobacter cloacae ATCC 13047	D5C6F6_ENTCC	ECL_01542	D5C6F7_ENTCC	ECL_01543	Rap2b (SMA2266)
Enterobacter hormaechei ATCC 49162	F5RYK9_9ENTR		F5RYK8_9ENTR		Rap2b (SMA2266)
Erwinia amylovora CFBP1430	D4I0Q7_ERWAC	EAMY_3018	D4I0Q6_ERWAC	EAMY_3017	Rap2a (SMA2265)
Erwinia tasmaniensis Et1/99	B2VH84_ERWT9	ETA_06210	B2VJE3_ERWT9	ETA_06220	Rap2a (SMA2265)
Escherichia coli B354	D6J6Z7_ECOLX	ECEG_03250	D6J6Z8_ECOLX	ECEG_03251	Rap2a (SMA2265)
Pantoea sp. Sc1	H8DNR2_9ENTR	S7A_11480	H8DNR1_9ENTR	S7A_11475	Rap2b (SMA2266)
Pseudomonas syringae pv. *syringae* B728a	Q4ZP52_PSEU2	Psyr_4040	Q4ZP51_PSEU2	Psyr_4041	Rap1b (SMA2262)
Salmonella Newport SL254	B4SV53_SALNS	SNSL254_A0303	B4SV54_SALN	SNSL254_A0304	Rap2a (SMA2265)
Salmonella Typhi CT18	Q8Z963_SALTI	STY0307	Q8Z964_SALTI	STY0306	Rap1a[2] (SMA2260)
Salmonella Typhimurium LT2	Q93IS4_SALTY	STM0277	Q8ZRL5_SALTY	STM0278	Rap2a (SMA2265)
Serratia odorifera DSM 4582	D4E4R6_SEROD		D4E4R5_SEROD		Rap1b (SMA2262)
Acinetobacter baumannii SDF	B0VVE3_ACIBS	p2ABSDF0033	B0VVE4_ACIBS	p2ABSDF0034	(Rap2a)[3] (SMA2265)
Serratia marcescens Db10	Ssp1	SMA2261	Rap1a	SMA2260	
Serratia marcescens Db10	Ssp2	SMA2264	Rap2a	SMA2265	

[1] Note that this is not the same protein as the one identified as a Tai4 protein in this organism by Russell *et al.*, 2012; that protein, ESA_03932, is an orphan Tai4 protein of the Rap2b type not immediately adjacent to Tae4

[2] STY0306 (SciQ) is approximately twice the size of other Rap proteins, resembling a fusion of two adjacent Rap1a proteins

[3] p2ABSDF0034 is shows only very weak sequence similarity with Rap2a

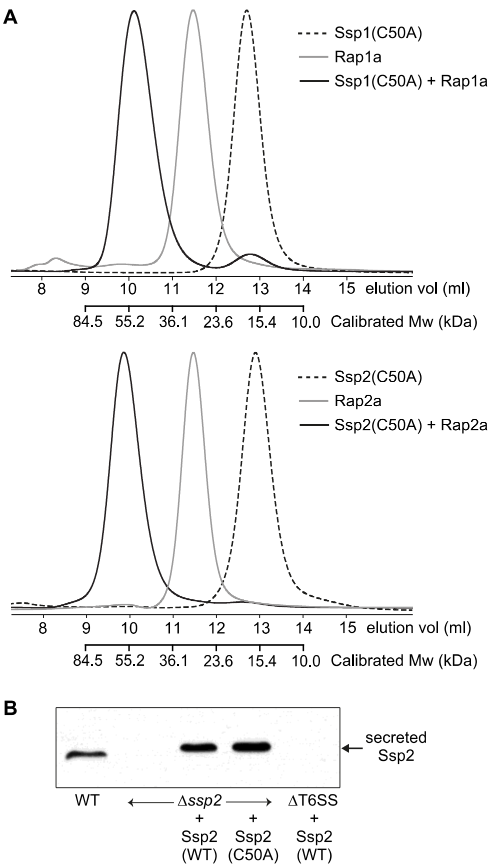

Supplementary Figure S1. Immunity protein binding and secretion is not impaired in Ssp1 and Ssp2 C50A mutants. (A) Size exclusion chromatography analysis of complex formation between Ssp1 (C50A) and Rap1a, top, or Ssp2 (C50A) and Rap2a, bottom. 10 nmol of the protein indicated, or of each protein in the case of the mixtures, was separated on a calibrated Superdex 75 10/300 GL column. (B) Immunoblot detection of Ssp2 in the secreted fraction of the strains indicated: wild type *S. marcescens* Db10 [WT]; mutant lacking Ssp2 [Δ*ssp2*]; Δ*ssp2* mutant carrying plasmids expressing wild type Ssp2 [+Ssp2(WT); pSC541] or the C50A mutant of Ssp2 [+Ssp2(C50A); pSC1230]; and a Type VI secretion system mutant [ΔT6SS] expressing wild type Ssp2.

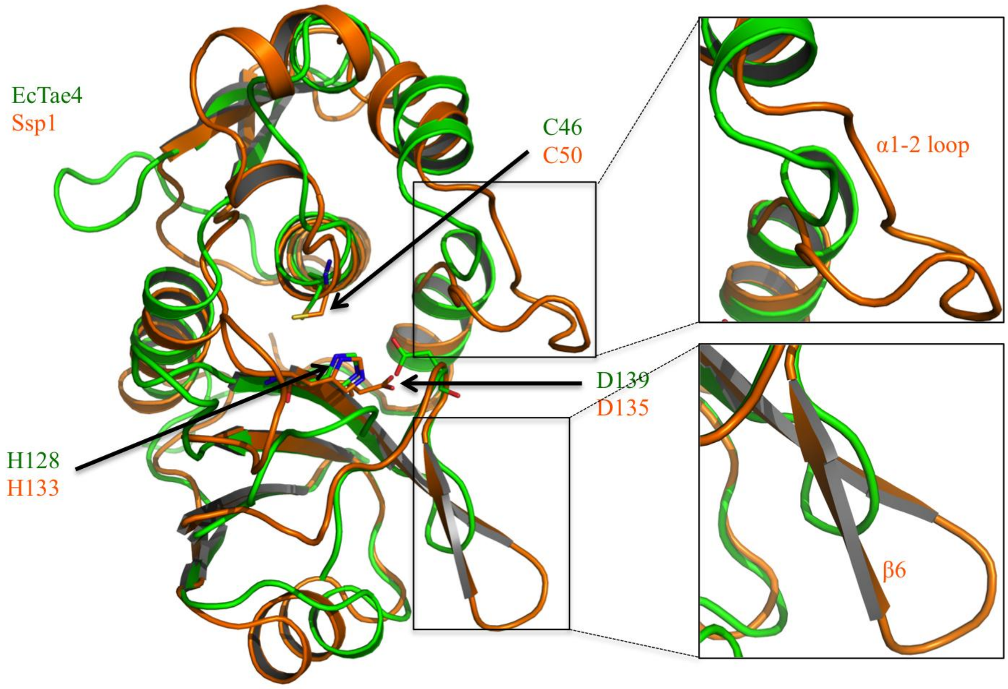

Supplementary Figure S2. Superimposition of Ssp1 (orange ribbon) and *Ec*Tae4 (green ribbon). The catalytic triads (histidine, cysteine and aspartate) are shown as sticks and divergent regions are shown in the boxes.

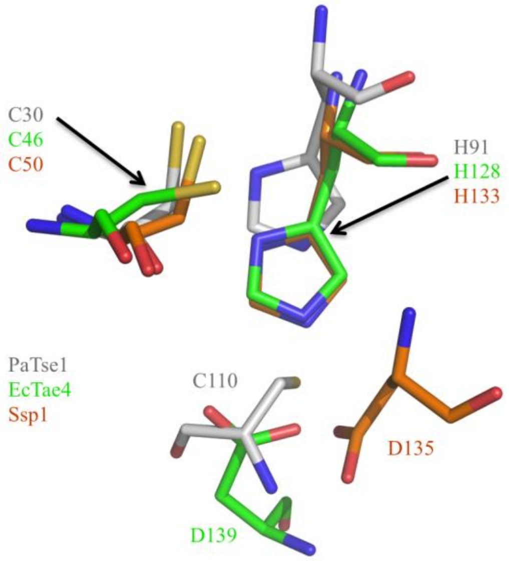

Supplementary Figure S3. Superimposition of the catalytic residues of Ssp1, *Pa*Tse1 and *Ec*Tae4. The color code is N blue, O red, S yellow then C positions for Ssp1 orange, *Pa*Tse1 grey and *Ec*Tae4 green.

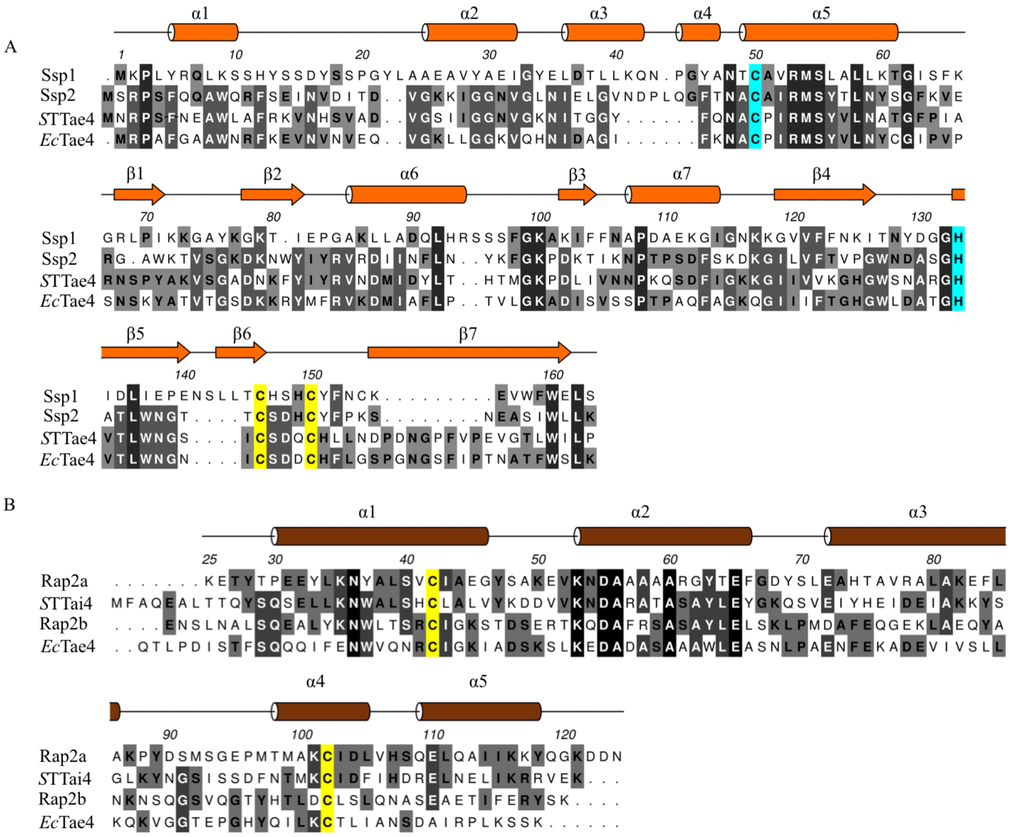

Supplementary Figure S4. Sequence alignment of Ssp1 and selected homologues. A. Structure-based sequence alignment highlights the conserved secondary-structure content (orange cylinders and sheets) in this group of four endopeptidase effectors. Residues involved in disulfide bond formation are coloured yellow. The catalytic histidine and cysteine residues are marked in cyan. The alignment was generated using ClustalW and the figure was prepared using *ALINE* (Bond & Schüttelkopf, 2009). B. An alignment of *S. marcescens* Rap2a with Tai4 from *E. cloacae* and *S.* Typhimurium. Sequence numbering and secondary structure has been drawn based on the Rap2a crystal structure (without signal peptide - brown). Strictly conserved residues in all four sequences are encased in black, conserved in two or three in shades of grey with the conserved cysteines involved in disulfide bond formation highlighted in yellow.

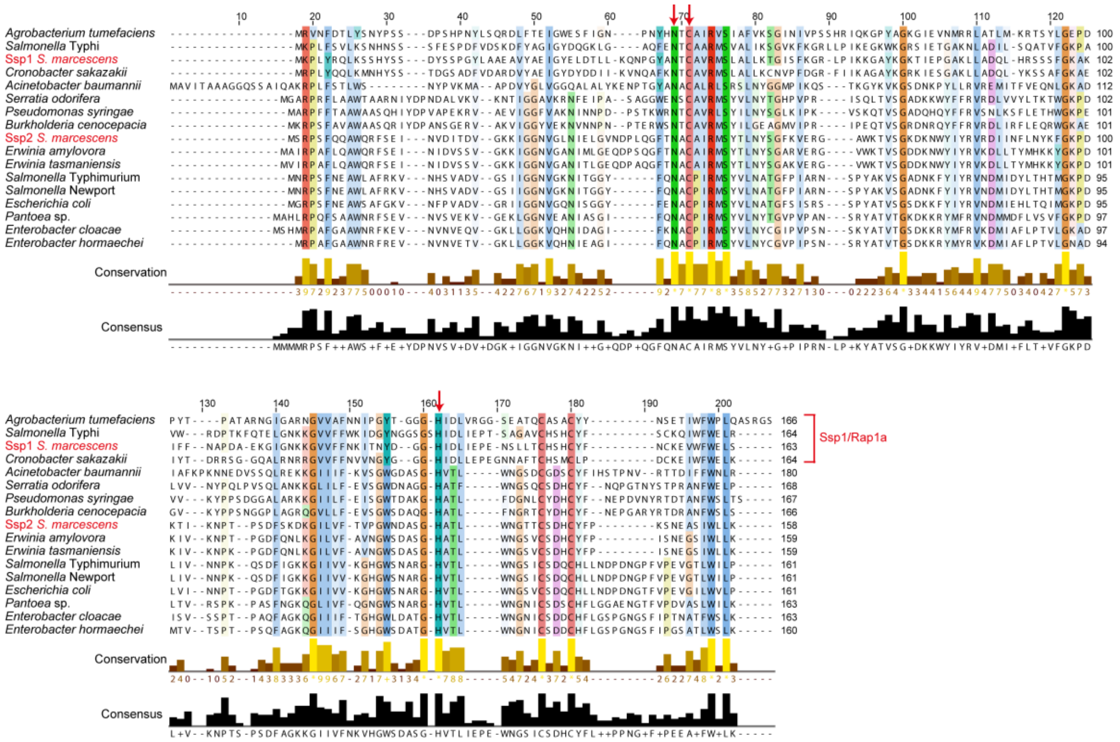

Supplementary Figure S5. Multiple sequence alignment of Ssp1 and Ssp2 with other Tae4 proteins. Alignment was generated using MUSCLE (Edgar, 2004) and visualised using Jalview (Waterhouse *et al.*, 2009). Red arrows show the amino acid residues forming the catalytic triad. The Ssp1-like proteins associated with Rap1a-type immunity proteins are also indicated. The Tae4 homologues are labelled by organism and their identities are as follows (UNIPROT identifier, genomic identifier): *Acinetobacter baumannii* (B0VVE3_ACIBS, p2ABSDF0033), *Agrobacterium tumefaciens* (Q7CUP8_AGRT5, Atu4347), *Burkholderia cenocepacia* (Q1BN86_BURCA, Bcen_4030), *Cronobacter sakazakii* (A7MQ14_CROS8, ESA_03935), *Enterobacter cloacae* (D5C6F6_ENTCC, ECL_01542), *Enterobacter hormaechei* (F5RYK9_9ENTR), *Erwinia amylovora* (D4I0Q7_ERWAC, EAMY_3018), *Erwinia tasmaniensis* (B2VH84_ERWT9, ETA_06210), *Escherichia coli* (D6J6Z7_ECOLX, ECEG_03250), *Pantoea* sp. (H8DNR2_9ENTR, S7A_11480), *Pseudomonas syringae* (Q4ZP52_PSEU2, Psyr_4040), *Salmonella enterica* serovar Newport (B4SV53_SALNS, SNSL254_A0303), *Salmonella enterica* serovar Typhimurium (Q93IS4_SALTY, STM0277) *Salmonella enterica* serovar Typhi (Q8Z963_SALTI, STY0307), *Serratia odorifera* (D4E4R6_SEROD). See also Supplementary Table S1.

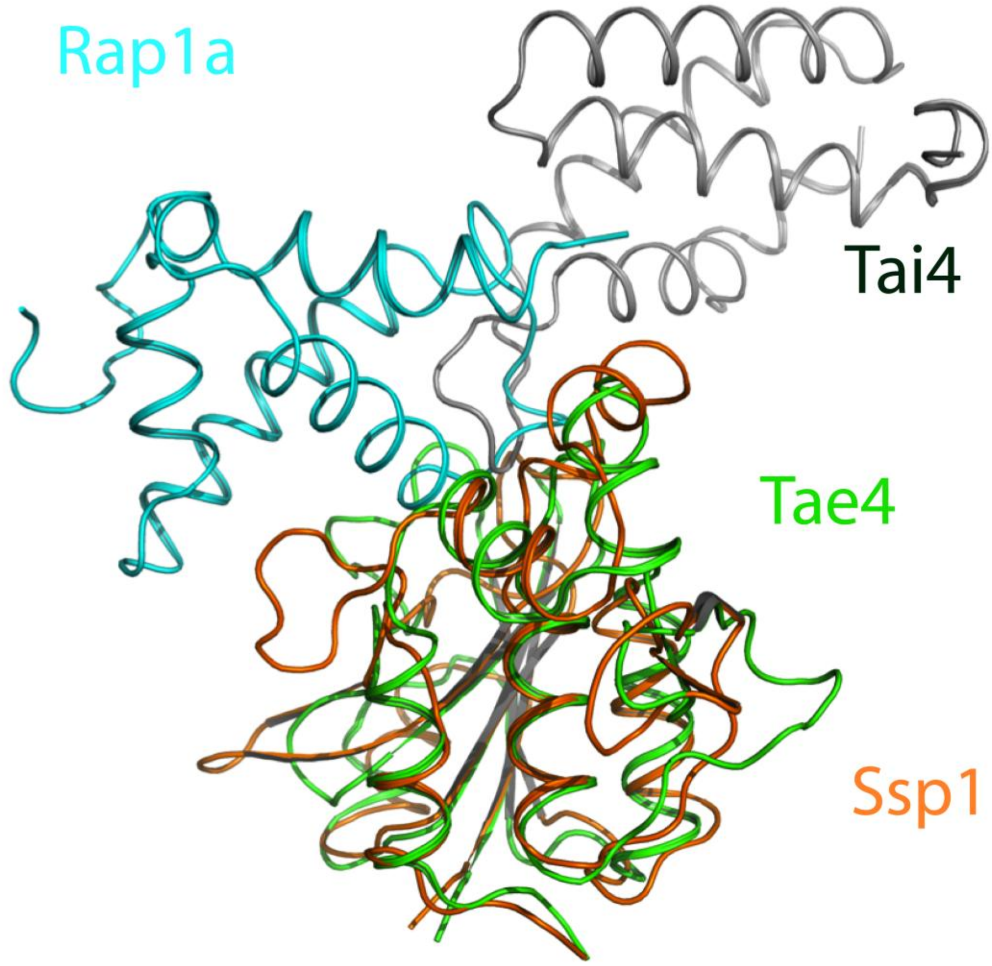

Supplementary Figure S6. Distinct placement of the immunity proteins inhibits different peptidoglycan endopeptidases. Four polypeptides are shown in ribbon style; Ssp1 is colored orange, *Ec*Tae4 is green, one subunit of *Ec*Tai4 is grey and one subunit of Rap1a is cyan. A least-squares overly of Ssp1 and Tae4 was carried out and this then highlights the different positions that the immunity proteins occupy when they bind and inhibit their cognate effector.
